# Supplementary material for: Sex Differences in Salmonellosis Incidence Rates—An Eight-Country National Data-Pooled Analysis
Source: J Clin Med. 2021 Dec 9;10(24):5767. doi: 10.3390/jcm10245767 (PMC8708425; doi:10.3390/jcm10245767)
Supplement: Supplementary file 1 [file jcm-10-05767-s001.zip › jcm-1478374-supplementary.pdf]

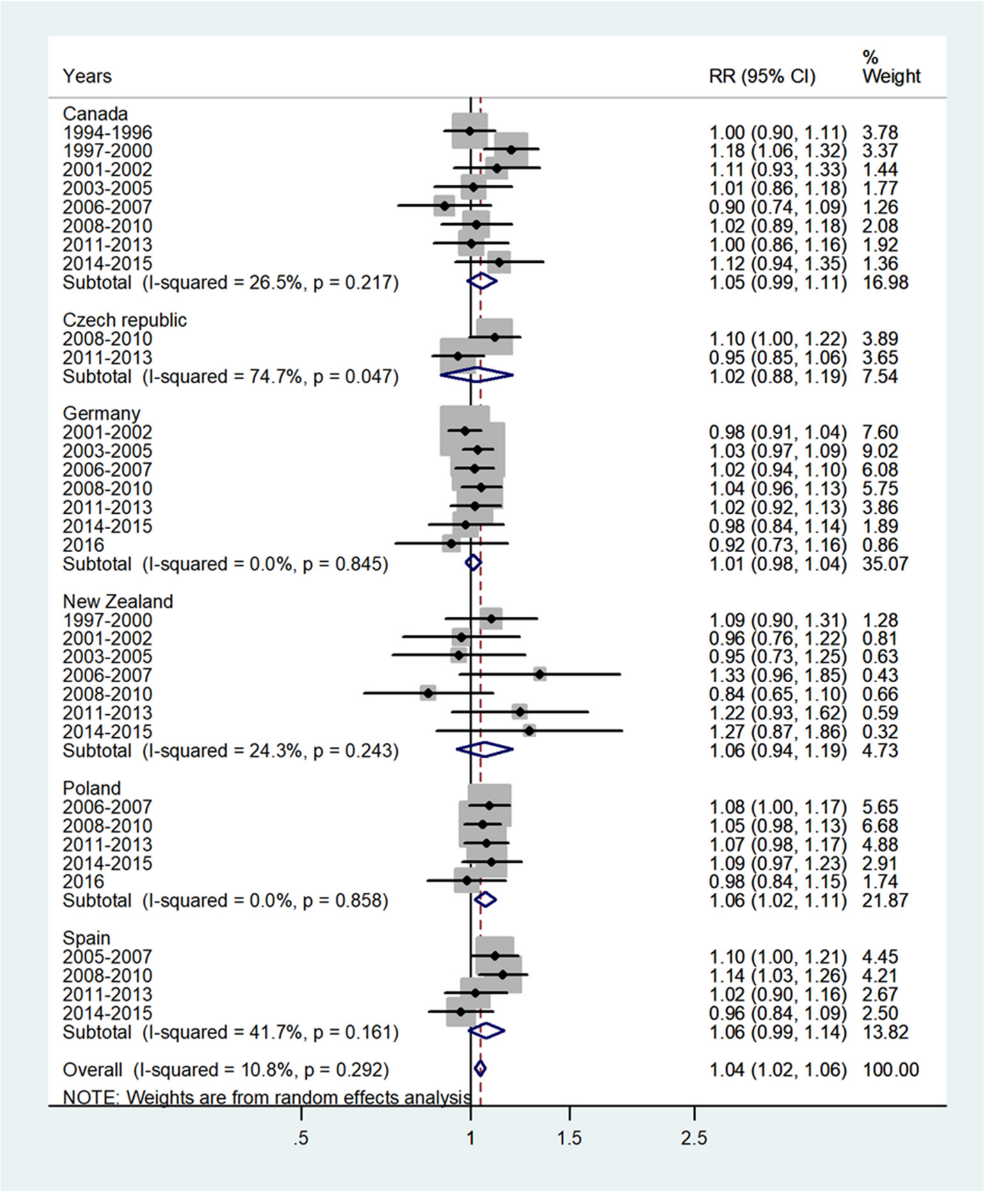

Figure S1. Male: female salmonellosis incidence rate ratios (RR) for different years and countries at age <1.

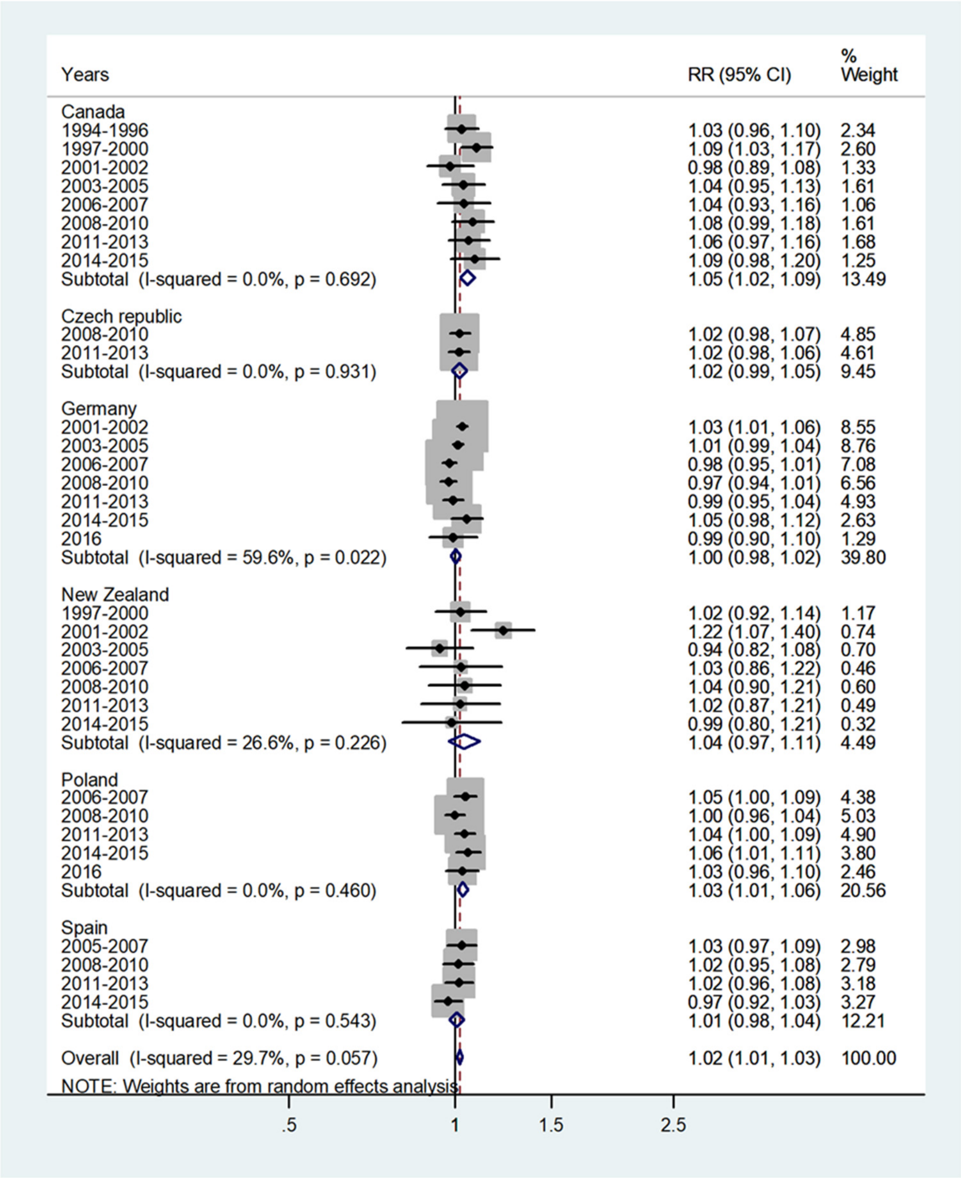

Figure S2. Male: female salmonellosis incidence rate ratios (RR) for different years and countries at age 1-4.

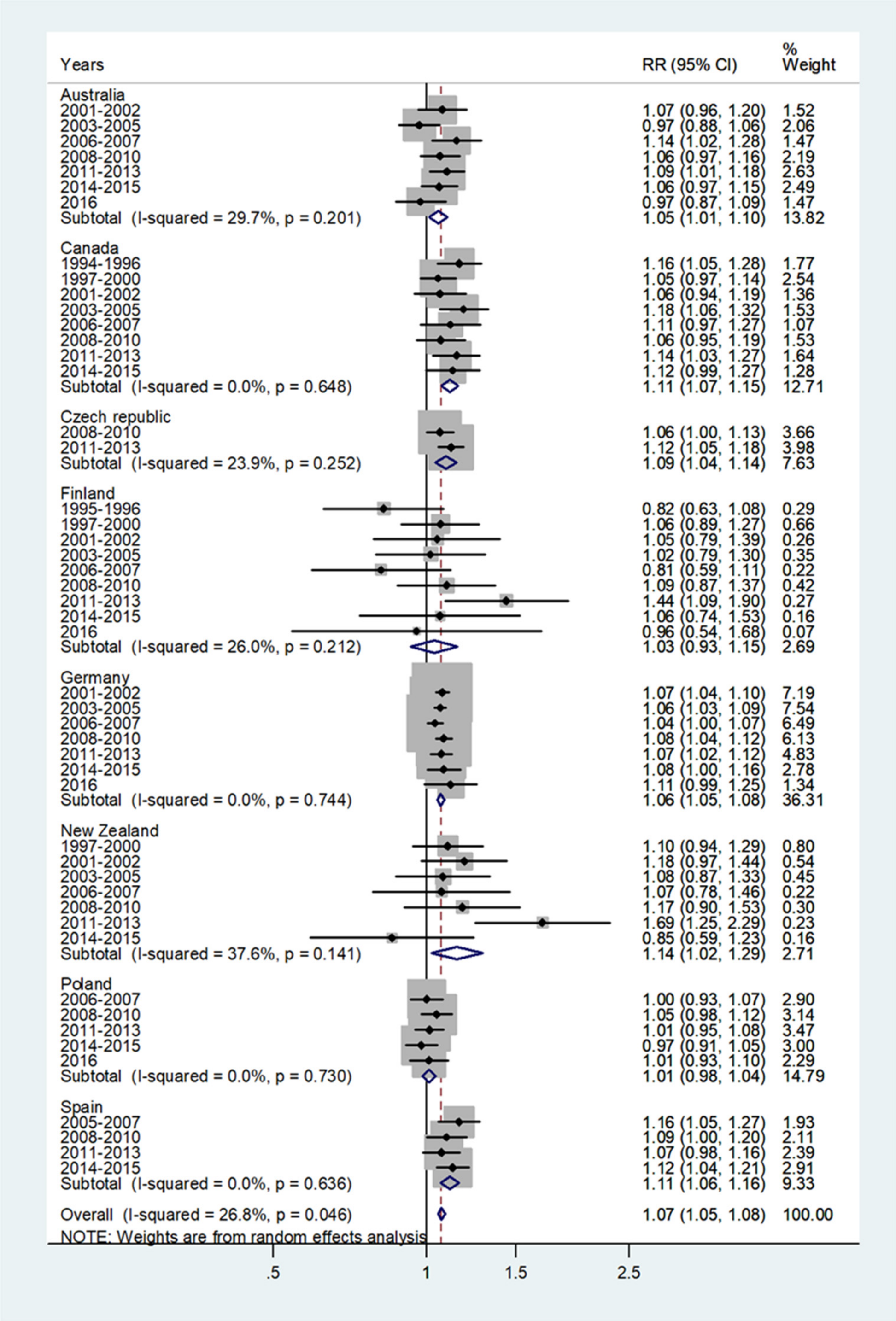

Figure S3. Male: female salmonellosis incidence rate ratios (RR) for different years and countries at age 5-9.

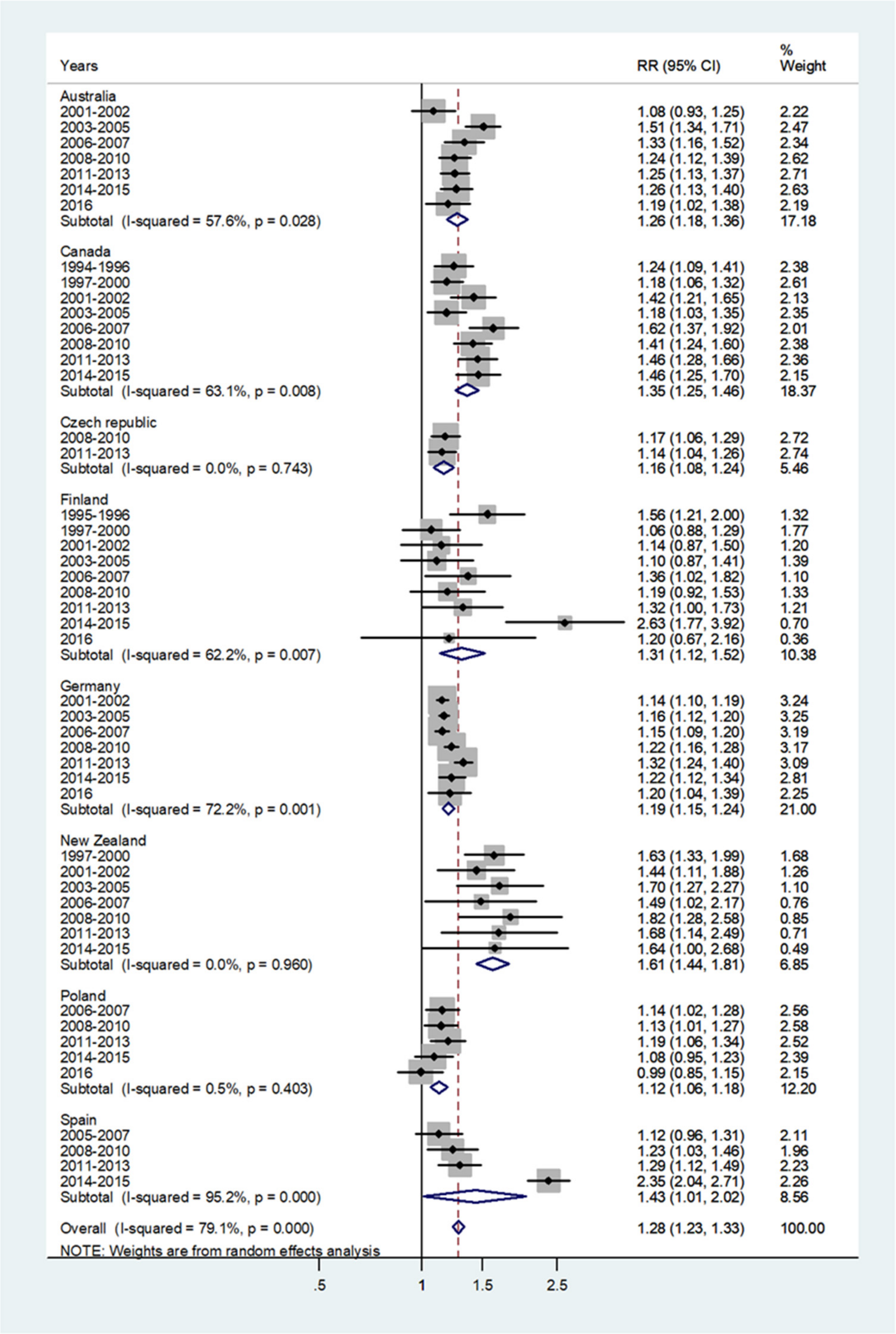

Figure S4. Male: female salmonellosis incidence rate ratios (RR) for different years and countries at age 10-14.

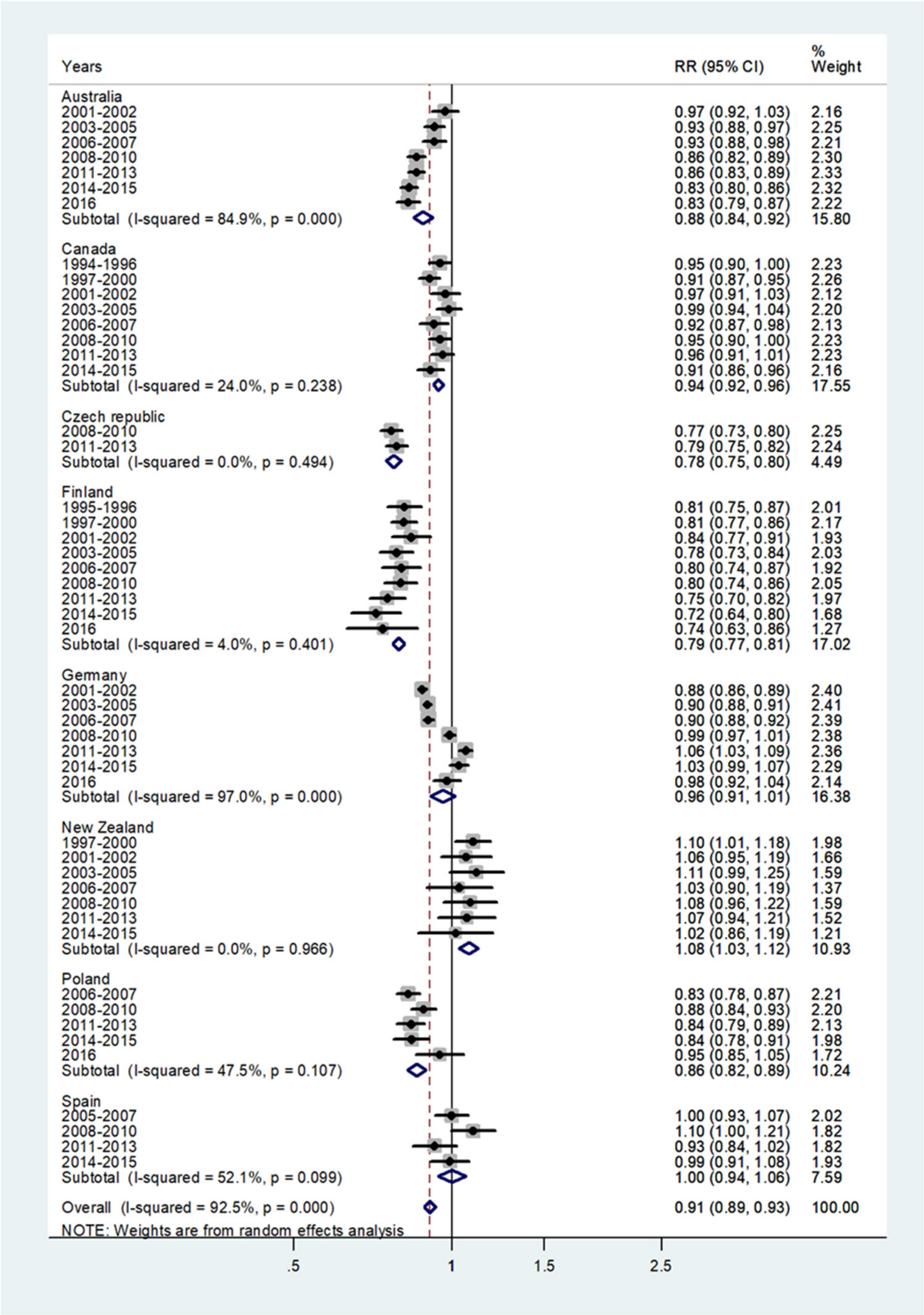

Figure S5. Male: female salmonellosis incidence rate ratios (RR) for different years and countries at age 15-39/15-44.

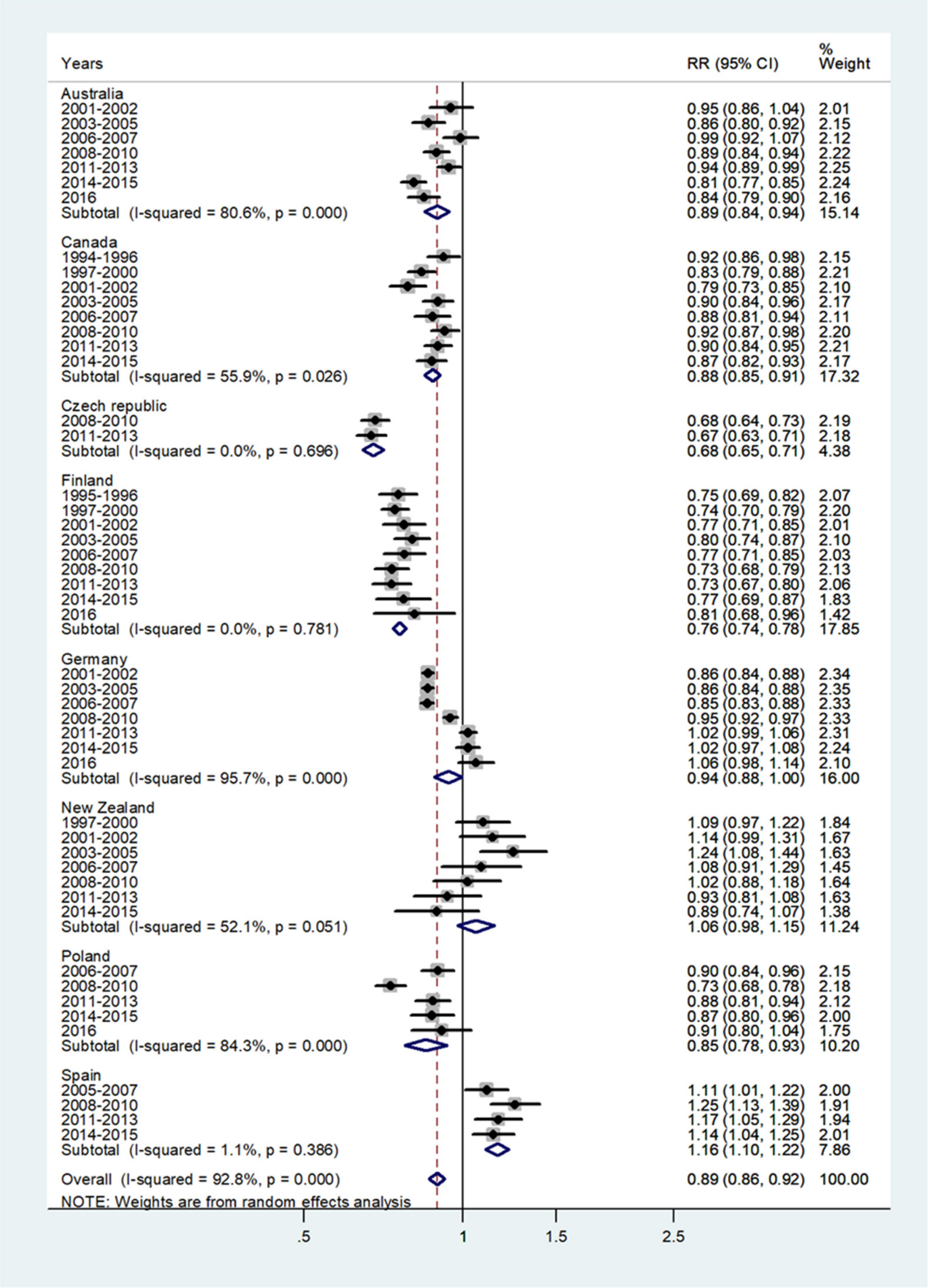

Figure S6. Male: female salmonellosis incidence rate ratios (RR) for different years and countries at ages 40-59/45-64.

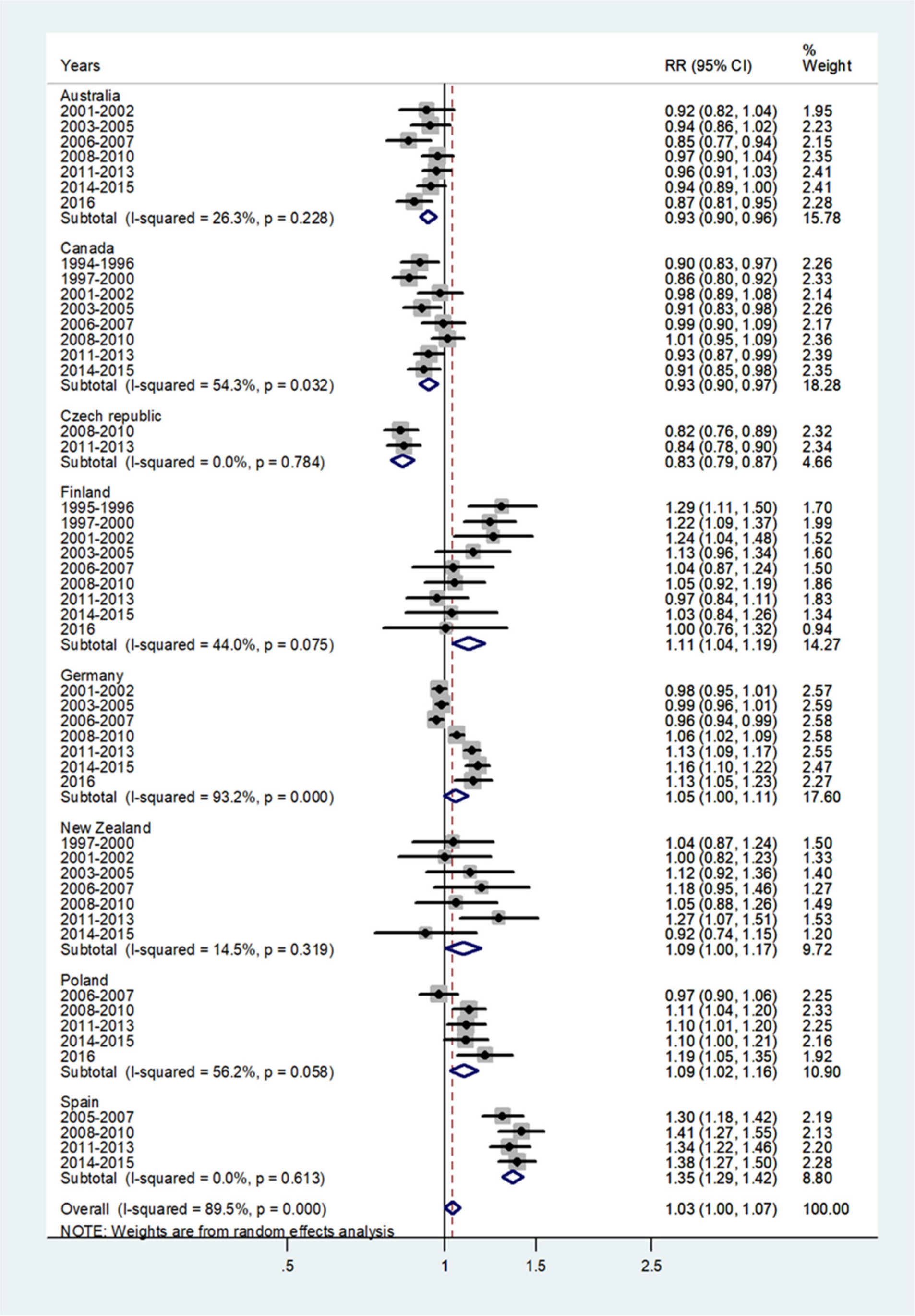

Figure S7. Male: female salmonellosis incidence rate ratios (RR) for different years and countries at ages 60+/65+.
